# Near 100% ethene selectivity achieved by tailoring dual active sites to isolate dehydrogenation and oxidation

**DOI:** 10.1038/s41467-021-25782-2

**Published:** 2021-09-14

**Authors:** Chaojie Wang, Bing Yang, Qingqing Gu, Yujia Han, Ming Tian, Yang Su, Xiaoli Pan, Yu Kang, Chuande Huang, Hua Liu, Xiaoyan Liu, Lin Li, Xiaodong Wang

**Affiliations:** 1 CAS Key Laboratory of Science and Technology on Applied Catalysis, Dalian Institute of Chemical Physics, Chinese Academy of Sciences, Dalian, People’s Republic of China; 2 University of Chinese Academy of Sciences, Beijing, People’s Republic of China; 3 Max Planck Institute for Chemical Physics of Solids, Dresden, Germany

**Keywords:** Catalyst synthesis, Heterogeneous catalysis, Chemical engineering

## Abstract

Prohibiting deep oxidation remains a challenging task in oxidative dehydrogenation of light alkane since the targeted alkene is more reactive than parent substrate. Here we tailor dual active sites to isolate dehydrogenation and oxidation instead of homogeneously active sites responsible for these two steps leading to consecutive oxidation of alkene. The introduction of HY zeolite with acid sites, three-dimensional pore structure and supercages gives rise to Ni^2+^ Lewis acid sites (LAS) and NiO nanoclusters confined in framework wherein catalytic dehydrogenation of ethane occurs on Ni^2+^ LAS resulting in the formation of ethene and hydrogen while NiO nanoclusters with decreased oxygen reactivity are responsible for selective oxidation of hydrogen rather than over-oxidizing ethene. Such tailored strategy achieves near 100% ethene selectivity and constitutes a promising basis for highly selective oxidation catalysis beyond oxidative dehydrogenation of light alkane.

## Abstract (web summary)

It is important but challenging to prohibit deep oxidation of alkene in oxidative dehydrogenation of light alkane. Here, dual active sites are tailored to isolate dehydrogenation and oxidation thus achieving superior ethene selectivity.

## Introduction

Oxidation catalysis plays a pivotal role in establishing green and sustainable chemical applications and contributes approximately 30% of total production in the chemical industry, such as selective oxidation of methanol to formaldehyde, propene to acrolein, butane to maleic anhydride, and ethene to ethylene epoxide, as well as the production of key pharmaceutical intermediates^1^. Unfortunately, a general and long-standing scientific conundrum is over-oxidation of desired product which is more reactive than parent substrate, impeding the industrialization process of numerous oxidation reactions with game-changing industrial value and economic benefits. Hence, selectively oxidative heterogeneous catalysis to restrain over-oxidation is significant but challenging whether for fundamental studies or practical applications^2–5^.

The oxidative dehydrogenation of light alkanes to olefins (ODH) which are important building blocks for a handful of industrial processes is a well-known example of such a challenging reaction that is a promising alternative to the current industrial practice of steam cracking with no thermodynamic limitation, coke formation, and large CO_2_ emission but difficult to realize commercialized utilization impeded by the liable deep oxidation^6–9^. This was because alkene is facile to be further oxidized before its desorption or re-adsorbed on the active sites of dehydrogenation (usually oxygen species) according to the Mars–van Krevelen mechanism^1,10,11^ which resulted from its higher affinity and reactivity than alkane to most surfaces particularly for those of V- and Ni-based catalysts which have been studied most for ODH^12–17^. Extensive studies have contributed to manipulating the chemical environment of active oxygen species to decrease its insertion as large extent as possible concurrently with activation for C−H bond in alkane. It was reported that supporting V_2_O_5_ and NiO with smaller clusters on the inert oxides such as SiO_2_ and Al_2_O_3_ led to the decrease in the reactivity of active lattice oxygen species to alkene thereby its further oxidation was alleviated, which stemmed from increased metal−oxygen bond strength. However, the activation for alkane declined^18–20^. Multiple metals doped vanadium oxide, e.g. Mo−V−Te−Nb mixed oxides were also reported to exhibit high ethene selectivity, which could be attributed to the V=O sites or redox-active O^−^ radical sites generated by the reduction of Te^4+^ to Te^0^ with low reactivity to ethene^7,8,21^. Recently, hexagonal boron nitride (BN) attracted continuous interest thanks to its unexpected olefin selectivity, which originated from the stabilization of propyl species by the nitroxyl radical in the active sites of >B−O−O−N< rather than highly reactive propyl radicals thus suppressing over-oxidation^22,23^. In addition, decreasing the number and restricting the local availability of active but nonselective such as electrophilic oxygen species O^−^ could also increase alkene selectivity induced by the decrease in the probability of continuous oxidation for various metals with higher valence doped NiO materials^24–29^. It was further clarified that isolated electrophilic oxygen species played a positive role for high ethene selectivity resulted from its effective breakage of C−H bonds of ethane to selectively produce ethylene with easy desorption^30,31^. Although great effort has been made, over-oxidation remains not to be completely avoided probably due to the difficulty to manipulate identical active sites such as oxygen species responsible for both dehydrogenation and oxidation resulting in facile further oxidation of alkene on it particularly when conversion increases. Consequently, it can be feasible to avert over-oxidation that dehydrogenation and oxidation occur on different active sites.

Herein, strategically tailoring dual Ni active sites is proposed by utilizing the acid sites, three-dimensional pore structure, and supercages of HY zeolite to anchor Ni^2+^ Lewis acid sites (LAS) and confine NiO clusters to isolate dehydrogenation and oxidation by lattice oxygen, respectively^2,32–36^. As a result, this strategy avoids consecutive oxidation and achieves near 100% C_2_H_4_ selectivity at a wider range of conversion compared with those reported in the previous work^37–40^. Experiments and theory studies indicate that such tailored dual active sites realizing isolated dehydrogenation and oxidation play a crucial role in avoiding over-oxidation of ethene.

## Results

### Catalysts preparation and characterization

**Fig. 1:**
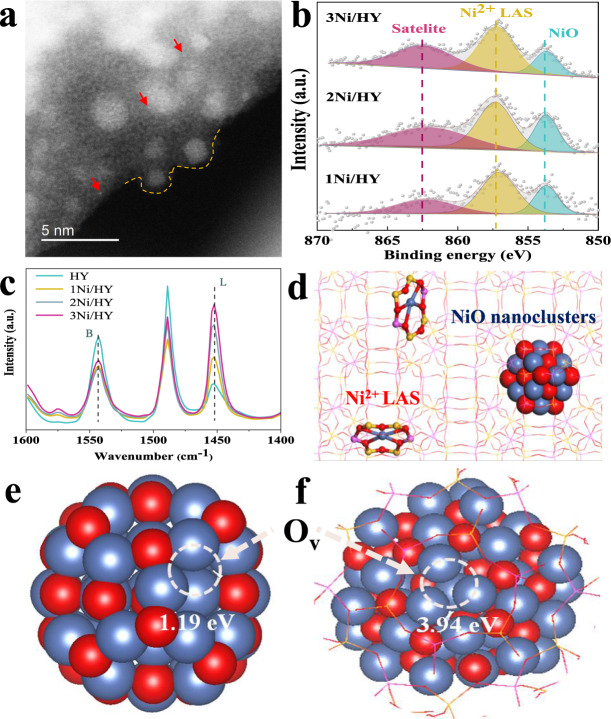
Characterizations of the *x*Ni/HY. **a** AC-TEM image of 3Ni/HY sample. **b** XPS spectra of fresh *x*Ni/HY. **c** Pyridine adsorption FT-IR spectra of *x*Ni/HY, where Brønsted (B) and Lewis (L) acid sites are observed at around 1540 and 1450 cm^−1^, respectively. **d** Scheme of the proposed local structure of Ni-modified HY. DFT calculation models and results of **e** 1 nm NiO cluster and **f** that confined inside zeolite. (Ov stands for oxygen vacancy).

The *x*Ni/HY were prepared with succinct and effective wet impregnation to create dual active sites to satisfy the need of isolating dehydrogenation and oxidation. A series of characterization was carried out to verify if HY introduced gave rise to dual Ni active sites. Firstly, high-angle annular dark-filed scanning transmission electron microscopy (HAADF-STEM) combined with energy dispersive spectrometer (EDS) mappings showed that Ni species with the exclusive valence state of +2 proved by Ni k-edge X-ray absorption near-edge spectrum (XANES) (Supplementary Fig. 2) were highly dispersed and well distributed with Si, Al and O elements with particle size ranging from 0.6 to 1.7 nm in *x*Ni/HY, which indicated that they entered the framework of HY for *x*Ni/HY (Supplementary Figs. 1, 3a−c). Aberration-corrected transmission electron microscope (AC-TEM) indicated the presence of two kinds of Ni species including the isolated cationic Ni^2+^ and NiO nanoclusters confined in HY framework in 3Ni/HY (Fig. 1a and Supplementary Fig. 4). X-ray photoelectron spectroscopy (XPS) presented that a peak at higher binding energy of about 857 eV attributed to isolated Ni^2+^ cations in HY with electron transferring to zeolite framework^41^ was also observed besides a peak at around 854 eV ascribed to NiO^42^ (Fig. 1b). It was reported that the introduction of metal species into zeolites would induce the generation of LAS by the substitution of Brønsted acid sites (BAS)^43^. Thus, the Ni species in the form of cations might be associated with Ni^2+^ LAS in HY. Pyridine adsorption FTIR spectra was performed to study Ni^2+^ LAS in HY. Three peaks of 1547, 1490, and 1455 cm^−1^ were seen (Fig. 1c) for HY, which corresponded to pyridine adsorption on BAS, BAS, and LAS, and LAS^44^. Significant increase of LAS and concurrent decrease of BAS content was observed for *x*Ni/HY and the variation extent becomes more prominent with Ni loadings, which suggested that Ni^2+^ LAS was generated by the substitution of protons of BAS in HY. This could be further supported by in situ FTIR wherein hydroxyl species associated with BAS in zeolites (3602 cm^−1^) was remarkably dropped for *x*Ni/HY samples compared with HY^45^ (Supplementary Fig. 5). UV−vis-NIR spectroscopy was employed to investigate the coordination environment of Ni cations in 3Ni/HY. A peak at around 425 nm attributed to *d*−*d* transition band of NiO had a visible blue shift for *x*Ni/HY in Supplementary Fig. 6, which indicated the decrease of coordination number of confined Ni species in HY compared with 6-coordinated octahedron structure within the NiO^11,41^. Density functional theory (DFT) calculation was then performed to determine the structure of Ni cations in HY. We compared several different local structures and found that Ni preferred sitting inside a six-member ring with two Al atoms and was four-coordinated by framework oxygen atoms (Supplementary Fig. 7). Other structures such as Ni sitting in different six-member or larger rings were less stable. Taken together, we successfully fabricated dual Ni active sites by HY introduced as presented in Fig. 1d, which might isolate dehydrogenation and oxidation thus avoiding over-oxidation. This was different from traditional ODH catalysts in which these two steps usually occurred on identical active sites leading to consecutive oxidation^10,46–48^. Considering the critical role of oxygen reactivity for the selectivity of alkene^2,10,49^, we calculated the energy of oxygen vacancy formation (*E*_OV_) of NiO nanoclusters confined in HY framework and bulky ones as a reference and the results indicated that Ni−O bond energy of NiO nanoclusters in HY framework was significantly higher than those of bulky NiO resulted from larger *E*_OV_ (Fig. 1e, f, and Supplementary Fig. 8a, b). This could be supported by the result of H_2_ temperature program surface reduction (H_2_-TPSR) on 3Ni/HY and a reference sample of 0.7Ni/silicalite-1 (0.7Ni/S-1) with NiO nanoclusters outside the framework of zeolite with a similar amount (Supplementary Table 1) and particle size (Supplementary Fig. 9) where reduction temperature for NiO nanoclusters in 3Ni/HY was significantly higher than those in 0.7Ni/S-1 (Supplementary Fig. 10). The design of dual Ni active sites with decreased oxygen reactivity of NiO nanoclusters confined in HY framework would be favorable for high selectivity to alkene likely resulted from the suppression of further oxidation of alkene.

### Performance of *x*Ni/HY with dual Ni active sites

**Fig. 2:**
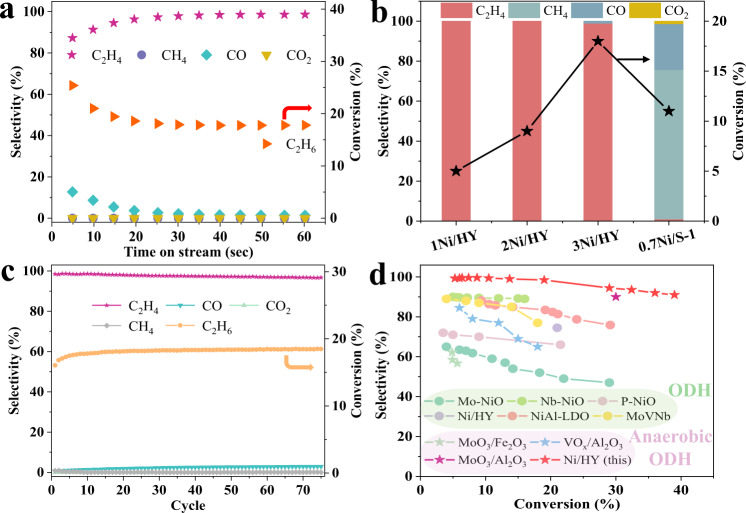
Performance evaluation of *x*Ni/HY for ethane ODH. **a** The conversion and selectivity vs. time for 3Ni/HY sample. **b** The performance of *x*Ni/HY and 0.7Ni/S-1. **c** Long-term stability test of 3Ni/HY during 75 cycles. **d** Performance comparisons^10, 31, 55–58^. It should be noted that Ni species were in the form of metallic Ni in Ni/HY of ref. ^47^, which was distinct from the dual active sites of Ni species in the present work.

The performance test was carried out under oxidative dehydrogenation and regeneration cycles without co-feeding O_2_. Figure 2a and Supplementary Fig. 11 show the evolution of gas product composition over *x*Ni/HY at 600 °C. Only C_2_H_4_ could be observed with a negligible amount of CO_2_ and CO (CO_*x*_) which accounted for C_2_H_4_ selectivity of 99% with the highest C_2_H_6_ conversion of 18% for 3Ni/HY during the overall oxidative dehydrogenation step. This indicated that our *x*Ni/HY catalysts with dual Ni active sites successfully prevented consecutive oxidation at a relatively wide range of conversion. Comparatively, 0.7Ni/S-1 with only NiO nanoclusters with a similar amount (Supplementary Table 1) and particle size (Supplementary Fig. 9) without Ni^2+^ LAS (Supplementary Fig. 12) exhibited completely overoxidation even at a lower conversion of about 10% (Fig. 2b and Supplementary Fig. 11), presenting that identical active site undertaking both dehydrogenation and oxidation in 0.7Ni/S-1 were facile to further oxidation of ethene on it. Moreover, 3Ni/HY outperformed most conventional oxidative dehydrogenation of ethane (ODHE) catalysts (Fig. 2d), indicating that the design of dual Ni active sites in *x*Ni/HY was beneficial to shun over-oxidation. It was worthwhile to note that oxygen consumption of *x*Ni/HY exceeded 30% (Supplementary Table 2), suggesting that lattice oxygen of NiO nanoclusters participated in the reaction and exhibited inferior activity for C_2_H_4_ oxidation probably due to its high bond strength (Fig. 1f and Supplementary Fig. 10). A long-term stability test was performed over 3Ni/HY for 75 (Fig. 2c) and 108 cycles (Supplementary Fig. 13). As shown in Fig. 2c, C_2_H_6_ conversion of about 18% and C_2_H_4_ selectivity of around 97% were almost identical during 75 cycles, indicating that the stability of performance of Ni confined HY catalysts for anaerobic ODHE. The characterization results of 3Ni/HY after reduction and long-term stability test (108 cycles) were provided in the Supplementary Figs. 14−16. It could be concluded that an identical amount of Ni^2+^ LAS and small NiO nanoclusters still residing in HY framework, i.e. unchanged dual Ni active sites, were responsible for the long-term stable performance of 3Ni/HY.

### Isolating dehydrogenation and oxidation

**Fig. 3:**
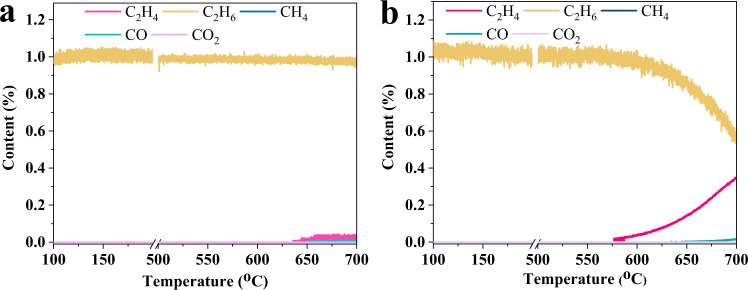
Ethane TPSR. **a** 0.7Ni@NaY and **b** 3Ni/HY, respectively.

**Fig. 4:**
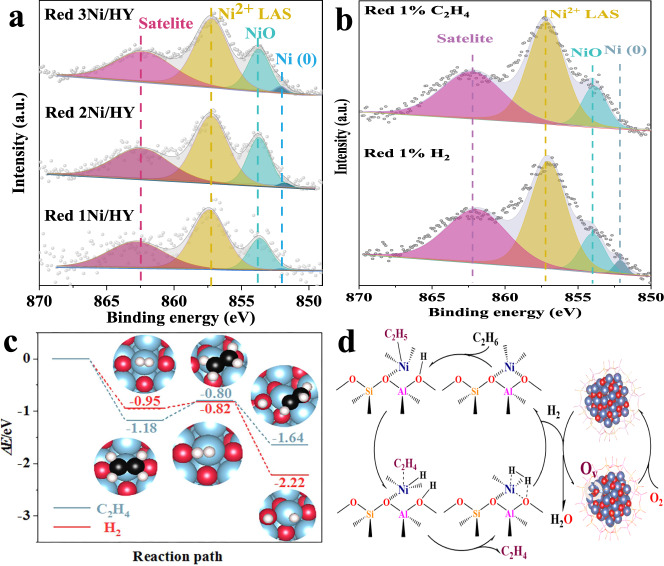
Reaction mechanism. Ni *2p*^3/2^ XPS spectra of **a** reduced *x*Ni/HY samples in reaction condition, and **b** 1%H_2_ and 1%C_2_H_4_ reduced 3Ni/HY. **c** Potential energies profiles for C_2_H_4_ and H_2_ dissociation pathways on 1 nm NiO cluster. Color index: Ni, blue, O, red, C, black, H, white. **d** Reaction mechanism proposed for anaerobic ODHE over Ni-modified HY.

In order to understand the superior selectivity of *x*Ni/HY, specific responsibility undertaken by Ni^2+^ LAS and NiO nanoclusters would be revealed. For this purpose, a reference sample of 0.7Ni@NaY was prepared (see the “Methods” section for details of its preparation) wherein the lack of BAS in NaY induced that Ni^2+^ LAS could not be generated by substituting BAS (Supplementary Fig. 17) thereby Ni species were present in the form of NiO nanoclusters confined in the framework of zeolite with a similar amount of 0.1 mmol/g and the average size of 1.2 nm (Supplementary Table 1 and Figs. 17, 18). Figure 3a, b shows ethane TPSR of 0.7Ni@NaY and 3Ni/HY. For 0.7Ni@NaY without Ni^2+^ LAS, C_2_H_6_ consumption could hardly be observed even the temperature reached 700 ^o^C accompanied with the formation of negligible C_2_H_4_ and no obvious CO_*x*_, indicating that NiO nanoclusters confined in NaY exhibited low activity for ODH of ethane. In comparison, C_2_H_4_ formed at a temperature of 580 °C without CO_*x*_ with consumption of C_2_H_6_ for 3Ni/HY, which strongly verified that Ni^2+^ LAS was responsible for the catalytic dehydrogenation of C_2_H_6_ to C_2_H_4_. This could be further confirmed by inferior C_2_H_6_ conversion of only 0.6% for 0.7Ni@NaY without Ni^2+^ LAS (Supplementary Fig. 20). The role of NiO nanoclusters in *x*Ni/HY for this reaction was then uncovered. XPS of reduced *x*Ni/HY showed that Ni^0^ with peak at around 852 eV^42^ formed (Fig. 4a) and the amount of lattice oxygen in O *1s* XPS decreased (Supplementary Fig. 21 and Supplementary Table 3). Moreover, the comparison of performance between 3Ni/HY-IE with only Ni^2+^ LAS (Supplementary Fig. 22, see the “Methods” section for details of its preparation and Ni^2+^ LAS amount in Supplementary Table 1) and 3Ni/HY with dual Ni active sites presented that 3Ni/HY exhibited remarkably higher C_2_H_6_ conversion than 3Ni/HY-IE did (Supplementary Fig. 23a) thus accounting for its larger C_2_H_4_ yield in spite of a slight decrease in C_2_H_4_ selectivity with an increase in C_2_H_6_ conversion (Supplementary Fig. 23b). Combining with the role of Ni^2+^ LAS, it could be inferred that lattice oxygen of NiO nanoclusters confined in the HY framework should be responsible for the improvement of selective conversion of ethane to ethene by oxidizing H_2_ rather than C_2_H_4_ formed from catalytic dehydrogenation of C_2_H_6_ on Ni^2+^ LAS. In order to confirm this, 3Ni/HY was subjected to C_2_H_4_ and H_2_ atmosphere under reaction temperature to study their oxidation. It could be seen that almost no consumption of C_2_H_4_ as well without CO_*x*_ signals were observed while H_2_ signals decreased remarkably with its consumption amount of 90 times to that of C_2_H_4_ (Supplementary Fig. 24). Moreover, Ni^0^ formed after H_2_ reduction but did not after C_2_H_4_ reduction (Fig. 4b), indicating that lattice oxygen of NiO nanoclusters selectively oxidized H_2_ instead of C_2_H_4_ formed. DFT calculations further demonstrated that the activation barrier of C_2_H_4_ dissociation into −C_2_H_3_ and −H was larger than that of H_2_ into −H on NiO nanoclusters (0.38 vs. 0.13 eV) with lower exothermic reaction energy (0.46 vs. 1.27 eV) in spite of its slightly larger adsorption energy (Fig. 4c and Supplementary Fig. 25), implying that NiO nanoclusters confined in HY framework were more tendentious to oxidize H_2_ than C_2_H_4_. This is considerably persuasive from the point of thermodynamics because of the increased Ni−O bond strength of NiO nanoclusters confined in HY framework (Fig. 1f). To verify it, we compared the performance of two double bed catalysts, namely one composed of 2.3Ni/HY-IE with only Ni^2+^ LAS (See the “Methods” section for details of its preparation and Ni^2+^ LAS amount in Supplementary Table 1) plus 0.7Ni/S-1 with only NiO nanoclusters outside S-1 zeolite and the other consisted of 2.3Ni/HY-IE plus 0.7Ni@NaY with only NiO nanoclusters confined inside Y zeolite. It could be seen that the double bed catalyst with 2.3Ni/HY-IE and 0.7Ni/S-1 suffered from complete over-oxidation whereas that with 2.3Ni/HY-IE and 0.7Ni@NaY exhibited nearly 100% C_2_H_4_ selectivity (Supplementary Figs. 26, 27), indicating that NiO nanoclusters confined in the framework with larger Ni−O bond strength than those outsides (Fig. 1e, f) was favorable for high selectivity to ethene resulted from the suppression of further oxidation of ethene formed on the Ni^2+^ LAS of 2.3Ni/HY-IE. This was supported by TPSR of ethene and H_2_ (co-feeding) on 0.7Ni/S-1 in which the consumption of large amount of ethene and conspicuous co-production of CO_*x*_ were observed (Supplementary Fig. 28). It was noted that the increase of H_2_ signals should be attributed to C_2_H_4_ cracking accompanied with CH_4_ formation. As a result, the fabrication of dual Ni active sites with reduced oxygen activity by HY introduced in the present work isolated dehydrogenation and oxidation, which prevented ethylene from successive oxidation at a wider range of conversion in turn achieving near 100% C_2_H_4_ selectivity at 18% C_2_H_6_ conversion for ODHE under anaerobic mode. The potential reaction pathway was depicted in Fig. 4d. Firstly, C_2_H_6_ adsorbed and activated on Ni^2+^ LAS by breaking C−H bonding of the methyl group leading to the formation of ethylnickel (Ni-CH_2_CH_3_) and BAS (Si−OH−Al). Subsequently, C_2_H_4_ desorbed by the β-hydrogen elimination to form metal hydride (Ni−H) and then Ni^2+^ LAS active sites restored inducing the formation of hydrogen^50^. Finally, lattice oxygen in NiO nanoclusters with increased Ni−O bond energy selectively removed hydrogen without over-oxidizing C_2_H_4_ (Fig. 4b and Supplementary Fig. 21) bringing about Ni^0^ species (Fig. 4a) which could be re-oxidized and regenerate to NiO nanoclusters by oxygen.

## Discussion

In summary, this work proposed and confirmed an efficient approach to prohibit over-oxidation, thus achieving superior ethylene selectivity for oxidative dehydrogenation of ethane in a redox manner. The high selectivity was contributed by the tailoring of dual Ni active sites to isolate dehydrogenation and oxidation in which Ni^2+^ LAS undertook the catalytic dehydrogenation of ethane to ethene, while NiO nanoclusters embedded in HY framework with enhanced Ni−O bond energy was capable to selectively transform hydrogen without over-oxidizing ethylene generated. Although this work is still plain only verified by oxidative dehydrogenation of ethane and a lot of work including more sufficient experiment validation, rational catalyst design, and process optimization needs to be done, such tailoring strategy to isolate dehydrogenation and oxidation on dual active sites toward avoiding consecutive oxidation opens a new avenue for selectively oxidative dehydrogenation of light alkanes and could be extended to multiple oxidation reactions that tendentiously suffer from the plaguing of overoxidation.

## Methods

### Preparation of Ni/HY with dual active sites

All the Ni modified HY samples (denoted as *x*Ni/HY, *x* indicated theoretical mass fraction of NiO) were synthesized by equal volume wetness impregnation method. For example, to prepare 3Ni/HY, Ni(NO_3_)_2_·6H_2_O (0.467 g) was dissolved in ultrapure water (3 mL) and then mixed with HY zeolite with SiO_2_/Al_2_O_3_ molar ratio of 5.4 (Nankai University). The resulting slurry was evenly dispersed by the aid of ultrasonic and then dried overnight at room temperature. After dried at 60 and 120 °C for 12 h, respectively, the samples were calcined at 600 °C for 4 h. Then they were ground and sieved into 40−60 mesh for reactivity evaluation.

### Synthesis of silicalite-1

Silicalite-1 (S-1, a MFI-type purely siliceous zeolite) was synthesized with a molar composition of 1.0 SiO_2_:0.2 TPAOH:46 H_2_O under conventional hydrothermal conditions at 443 K for 72 h^51^. Afterwards, the as-synthesized solids were centrifuged and washed several times with deionized water, then dried at 373 K for 12 h and calcined at 823 K for 6 h in air with heating rate 2 °C min^−1^ to remove the organics.

### Preparation of 0.7Ni/S-1

0.7Ni/S-1 was also prepared by the above impregnation procedure with Ni(NO_3_)_2_·6H_2_O.

### Synthesis of 2.3Ni/HY-IE and 3Ni/HY-IE

The 2.3Ni/HY-IE and 3Ni/HY-IE samples were synthesized by nickel exchange of the HY support using a template ion-exchange procedure^52^. Nickel exchange was performed at 348 K for 16 h using 200 ml of aqueous 0.007 and 0.01 M Ni(NO_3_)_2_ solution with 4 g solid support, respectively. The exchanged product was collected by filtration and washed with ultrapure water. The obtained catalyst was dried at 353 K for 12 h and then calcined at 773 K for 4 h.

### Synthesis of 0.7Ni@NaY

0.7Ni@NaY samples with confined NiO clusters within FAU zeolites were synthesized by the hydrothermal method in the absence of organic template agents and modified with the addition of base metal cations and protecting ligands to form confined NiO clusters^53^. Concretely, synthesis gels were prepared by first dissolving 0.65 g N-[3-(trimethoxysilyl)propyl]ethylenediamine (TPE; 98%, Sigma-Aldrich) and 0.2 g Ni(NO_3_)_2_·6H_2_O into ultrapure H_2_O (26.5 ml). NaAlO_2_ (2.34 g) and NaOH (3.77 g) were added sequentially while stirring (20 min). Colloidal silica (28.6 g; Ludox AS-30) was then added to this solution at ambient temperature and stirred for 2 h to prepare homogeneous synthesis gels (10 SiO_2_/1.0 Al_2_O_3_/4.3 Na_2_O/180 H_2_O/0.050 metal/0.20 ligand, molar ratios). The gels were sealed within 100 ml PTFE-lined stainless steel autoclave and heated at 393 K for 24 h without stirring to form Ni@NaY solids. The products were collected by filtration and washed by ultrapure water until to PH of 7−8, then dried at 373 K for 8 h and heated in flowing dry air to 623 K (at 2 °C min^−1^) and held for 3 h. The as-synthesized samples with well-defined FAU structure and about 1−2 nm confined NiO clusters without obviously NiO diffraction peaks and aggregation of Ni species outside zeolite frameworks (Supplementary Figs. 18, 19).

### Characterization of materials

The crystalline structure of samples was measured by PANalytical X’Pert-Pro powder X-ray diffractometer with Cu Kα monochromatized radiation (λ = 0.1541 nm, 40 kV, 40 mA). The scanned 2θ range was 10−80°. The NiO particle size and element distribution of samples before and after cycling were studied by scanning transmission electron microscopy (STEM) and energy dispersive spectroscopy (EDS), respectively, using a field-emission transmission electron microscope (JEOL JEM-2100F). The AC-HAADF-STEM analysis was performed on a JEOL JEM-ARM200F equipped with a CEOS probe corrector, with a guaranteed resolution of 0.08 nm. Before microscopy examination, the sample was ultrasonically dispersed in ethanol for 15–20 min, and then a drop of the suspension was dropped on a copper TEM grid coated with a thin holey carbon film. XPS measurement of the fresh and reduced samples was performed with a VG ESCALAB210 spectrometer equipped with an Al Ka (hm = 1486.6 eV) X-ray source. The binding energies were calibrated using the C *1s* peak at 284.3 eV as a reference. The Brønsted and Lewis acid sites (BAS and LAS) of samples were quantified by pyridine adsorption IR measurement. The content of BAS and LAS were obtained using molar extinction coefficients^54^. Actual NiO loadings were measured by inductively coupled plasma-optical emission spectrometry (ICP-OES) on an IRIS Intrepid II XSP instrument (Thermo Electron Corporation). The *d*−*d* transition of *x*NiO/HY (*x* = 1−3%) and bulky NiO were obtained by Ultraviolet–visible (UV−vis) absorption spectroscopy on a PerkinElmer Lambda 950 spectrometer. In situ IR spectra of dehydrated xNiO/HY (*x* = 0, 1, 3, 6) were analyzed by a Fourier transform infrared (FTIR) analyzer (Bruker, TENSOR27) at a transmission mode, equipped with a liquid N_2_-cooled mercury−cadmium−telluride detector. Dehydration was performed under vacuum at 773 K for 1 h. After cooling to 393 K, the −OH group signals of the samples were collected. The X-ray absorption fine structure (XAFS) at the K-edge of Ni was measured on beam line BL14W1 of the Shanghai Synchrotron Radiation Facility (SSRF), China. A double Si (111)-crystal monochromator was used for energy selection. The energy was calibrated by the Ni foil. The spectra were collected at room temperature under the transmission mode. The data were analyzed by the Athena software package.

### TPSR experiment

Ethane TPSR was performed in a fixed bed reactor loaded with 200 mg samples. The reaction temperature was monotonously increased to 700 °C from 100 °C at a rate of 5 °C min^−1^ in 1% C_2_H_6_/He (150 mL/min) and the outlet gas was detected by an on-line FTIR (Bruker, MATRIX-MG(HR)-01). H_2_ TPSR was performed in a fixed bed reactor loaded with 200 mg samples. The reaction temperature was monotonously increased to 900 °C from 100 °C at a rate of 10 °C min^−^^1^ in 10% H_2_/He (30 mL/min) and the outlet gas was detected by an on-line quadrupole MS (IPI, Model GAM200).

### H_2_ and C_2_H_4_ co-feeding experiment

The NiO cluster oxidation for H_2_ and C_2_H_4_ was studied in a fixed bed reactor with H_2_ and C_2_H_4_ co-feeding. The reaction temperature increased to 600 °C keeping 20 min in an inert atmosphere. Then, 1% H_2_-1% C_2_H_4_/He (30 mL/min) was forced into a reactor and kept that until no obvious consumption of H_2_ and C_2_H_4_. The consumption of hydrogen and ethene was quantified by an on-line quadrupole MS (IPI, Model GAM200).

### Reactive performance tests

Activity tests were performed in a quartz fixed-bed reactor with an inner diameter (ID) of 7.5 mm loaded with 1.0 g samples (40−60 mesh) under atmospheric pressure. The operation reaction temperature is typically 600 °C, monitored by using a K-type thermocouple. The whole reaction process is divided into two steps, which include isolated reduction and oxidation step. The reduction step was performed with a space velocity of 5100 h^−1^ in 10% ethane balanced in helium for 30 s. The samples were re-oxidized in the oxidation step by using 5% oxygen in helium with the flow rate of 30 mL/min for 5 min. In order to prevent the mixing between ethane and oxygen, the reactor is purged with helium (150 mL/min) for 2 min between the reduction and re-oxidation step. Detailed methodologies for IR quantification and oxygen consumption calculations were described in supplementary Materials.

### DFT calculations

All the oxygen vacancy formation energy was performed using the Vienna ab initio simulation package (VASP) with projector augmented-wave (PAW) method. For the relaxations of model structures of NiO clusters in vacuum and Zeolite, the exchange-correlation functional of general gradient approximation (GGA) type in the parameterization by Perdew-Burke-Ernzerhof (PBE) was adapt. The forces felt by each of the atoms were well converged below 0.02 eV·Å^−^^1^, and the energy convergence was set as 10^−^^5^ eV. The relaxation of NiO clusters in a vacuum was done in a cubic cell of 30 Å × 30 Å × 30 Å. For all calculations, Hubbard U type correction was adopted to address the electron correlation for the 3d electrons of Ni with an effective *U* value of 5 eV, the cutoffs of the wave function is 500 eV, and A k-mesh with the density of one point per ~0.03 Å^−^^3^ was generated using the Monkhorst−Pack method. The climbing image nudged elastic band (CI-NEB) method was employed to locate the transition states.

## Supplementary information

Supplementary Information

## Data Availability

The data that support the findings of this study are available from the corresponding author upon reasonable request.
