# Supplementary material for: Near 100% ethene selectivity achieved by tailoring dual active sites to isolate dehydrogenation and oxidation
Source: Nat Commun. 2021 Sep 14;12:5447. doi: 10.1038/s41467-021-25782-2 (PMC8440631; doi:10.1038/s41467-021-25782-2)
Supplement: Supplementary file 1 — Supplementary Information [file 41467_2021_25782_MOESM1_ESM.pdf]

Supplementary Information

Near 100% ethylene selectivity achieved by tailoring dual active sites to isolate dehydrogenation and oxidation

Chaojie Wang[1,2], Bing Yang[1], Qingqing Gu[1], Yujia Han[1,2], Ming Tian[1*], Yang Su[1], Xiaoli Pan[1], Yu Kang[3], Chuande Huang[1], Hua Liu[1,2], Xiaoyan Liu[1], Lin Li[1] & Xiaodong Wang[1*]

[1]CAS Key Laboratory of Science and Technology on Applied Catalysis, Dalian Institute of Chemical Physics, Chinese Academy of Sciences, 457 Zhongshan Road, Dalian 116023, People's Republic of China.

[2]University of Chinese Academy of Sciences, 19(A) Yuquan Road, Shijingshan District, Beijing 100049, People's Republic of China.

[3]Max Planck Institute for Chemical Physics of Solids, Dresden 01187, Germany.

[*]Corresponding author: E-mail: tm1982@dicp.ac.cn; xdwang@dicp.ac.cn.

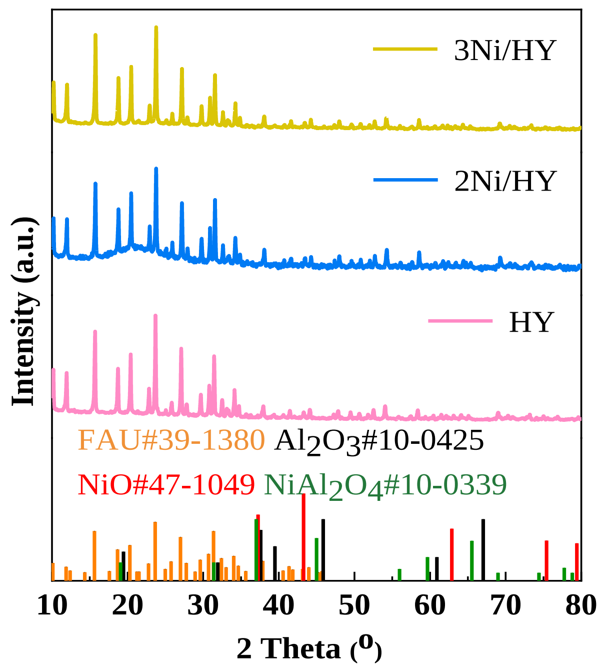

Supplementary Figure. 1 XRD patterns of as-prepared xNi/HY. XRD patterns showed that no peaks of nickel or nickel oxide except those attributed to FAU structure were observed, which should be due to high dispersion of Ni species in framework of HY or low Ni amount.

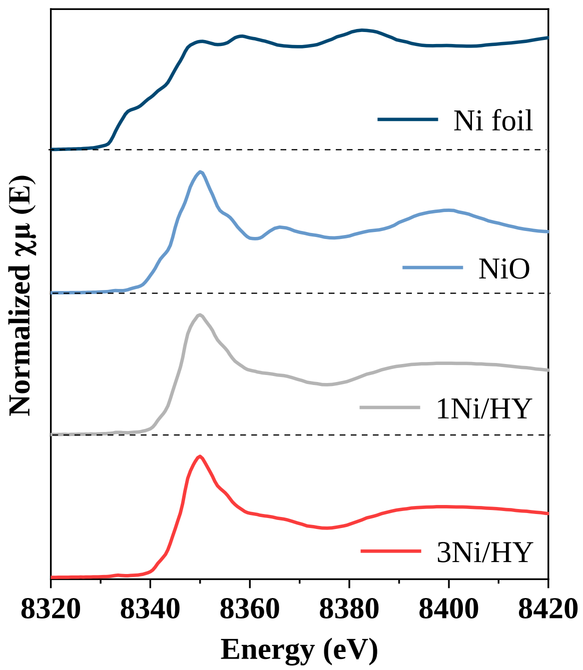

Supplementary Figure. 2 Normalized Ni k-edge X-ray absorption near-edge spectrum (XANES) of xNi/HY.

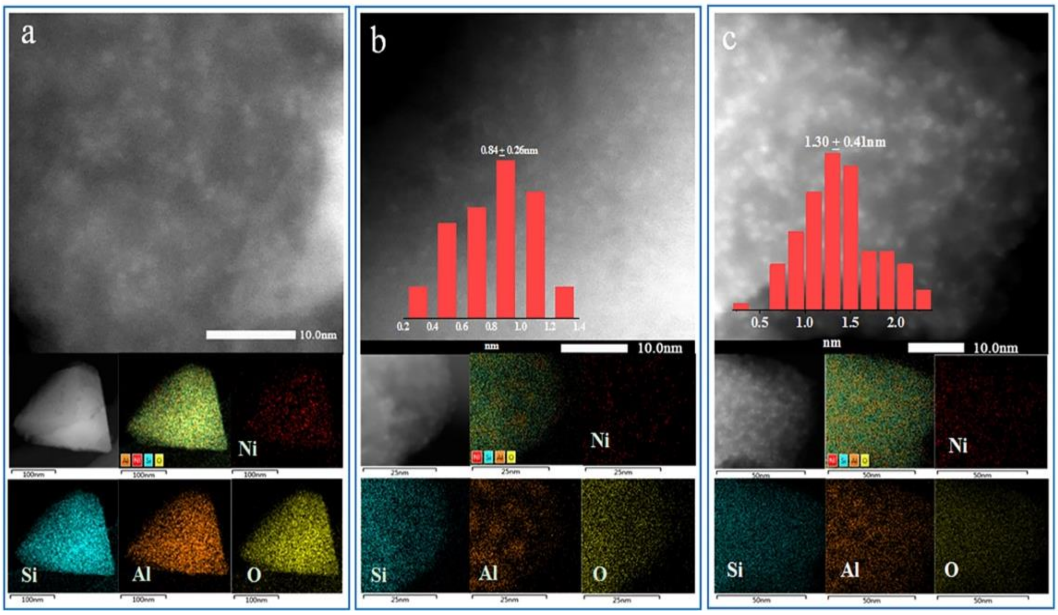

Supplementary Figure. 3 a-c HAADF-STEM images and EDS mappings of the fresh xNi/HY (x=1, 2, and 3), respectively.

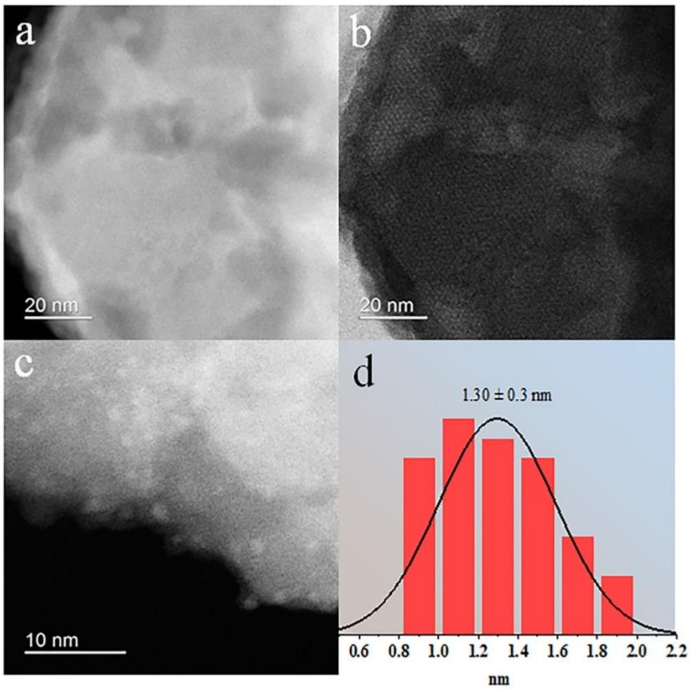

Supplementary Figure. 4 Aberration-corrected Transmission Electron Microscope images of 3Ni/HY. AC-TEM results showed that the presence of two kinds of Ni species including the isolated cationic Ni and NiO nanoclusters confined in HY framework and no aggregation NiO particles outside zeolites framework.

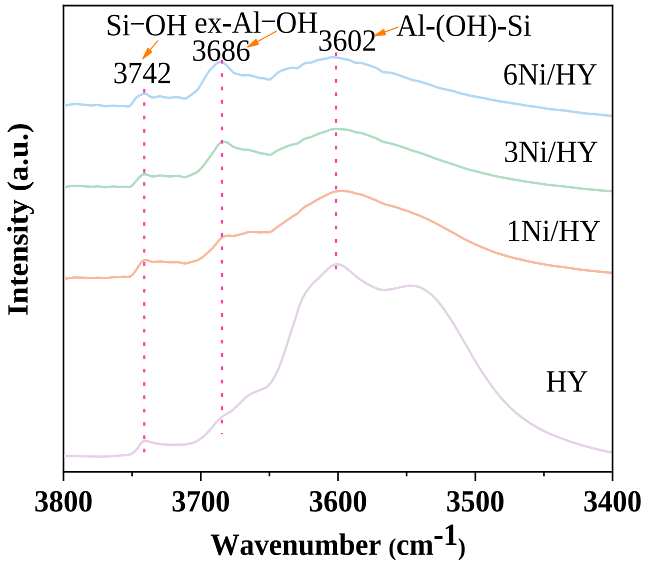

Supplementary Figure 5. In situ IR spectra of dehydrated xNi/HY collected at 383 K. Dehydration was performed at 773 K for 1 h.

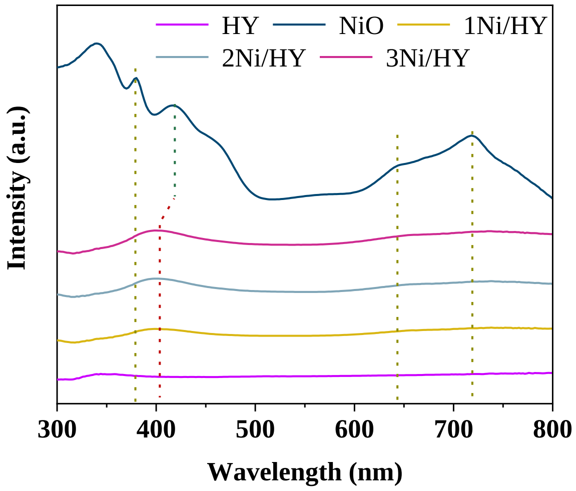

Supplementary Figure. 6 UV−vis−NIR absorption spectra of NiO, HY and nickel-containing HY zeolites.

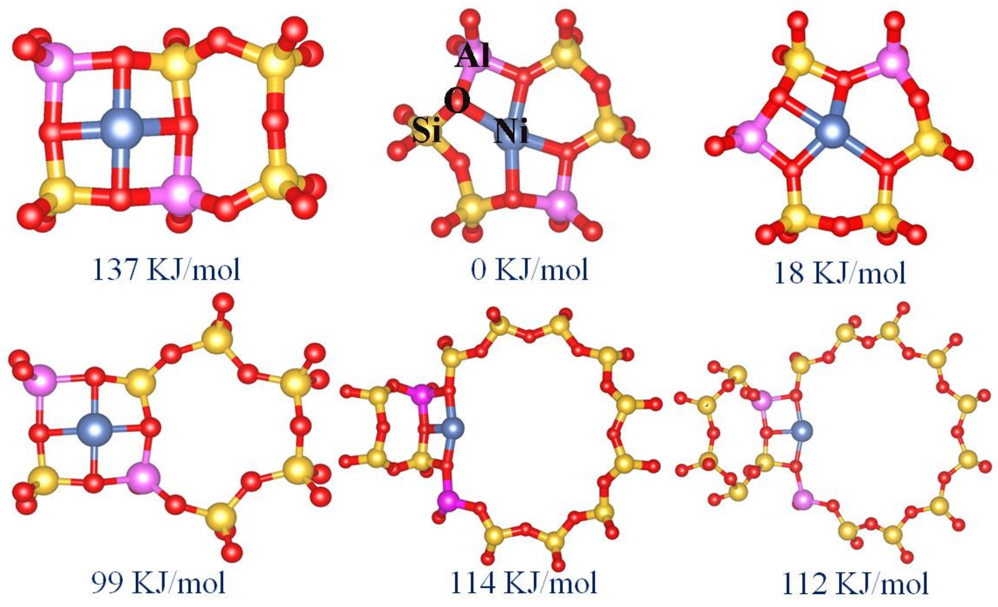

Supplementary Figure. 7 Possible coordination structures with corresponding relative energy of Ni^{2+} LAS in Ni-modified HY.

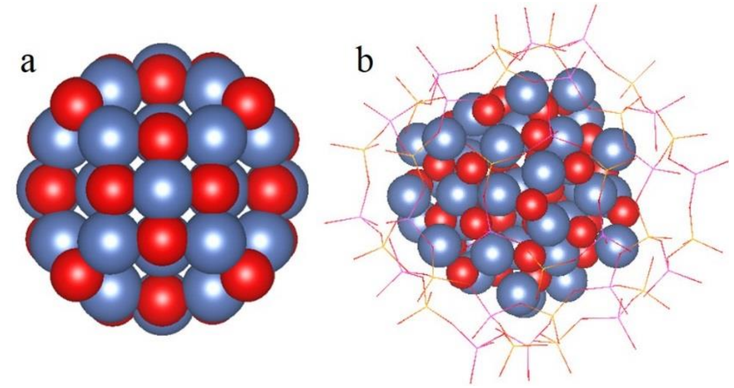

Supplementary Figure. 8 DFT computational models of **a** 1nm NiO cluster and **b** that encapsulated inside cage of zeolite.

Given the pore and cage surface of zeolite is oxygen enrichment that cause electrostatic repulsion between framework oxygen of zeolite and dangling oxygen of NiO cluster with an oxygen-enriched structure leading to the increasement of system energy, therefore, we adopted the nickel- dangling NiO cluster structure to model NiO-modified zeolite. The interaction between NiO clusters and Zeolite is constructed by balancing charge of BAS in which hydrogen atom is removed. The lattice oxygen vacancy formation energy can be defined as the following equations:

$$\Delta E_{formation,\ vac}=E_{defective}+0.5E_{O2}-E_{perfect} \quad (1)$$

Where $E_{perfect}$ denotes the perfect crystal energy of NiO cluster and NiO-modified Zeolite. $E_{defective}$ denotes its surface with one oxygen vacancy. E_{O2} denotes the calculated oxygen energy which is in the vacuum phase. The DFT computational models were displayed in **Supplementary Figure 8**.

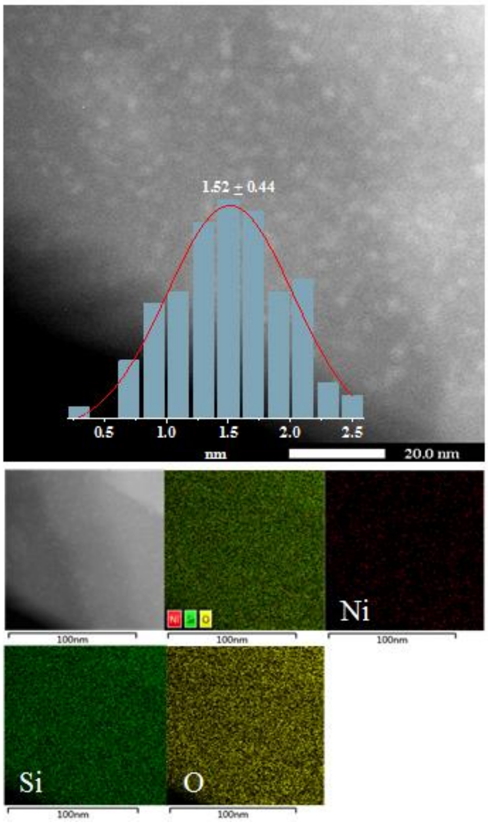

Supplementary Figure. 9 HAADF-STEM images and EDS mappings of 0.7Ni/S-1.

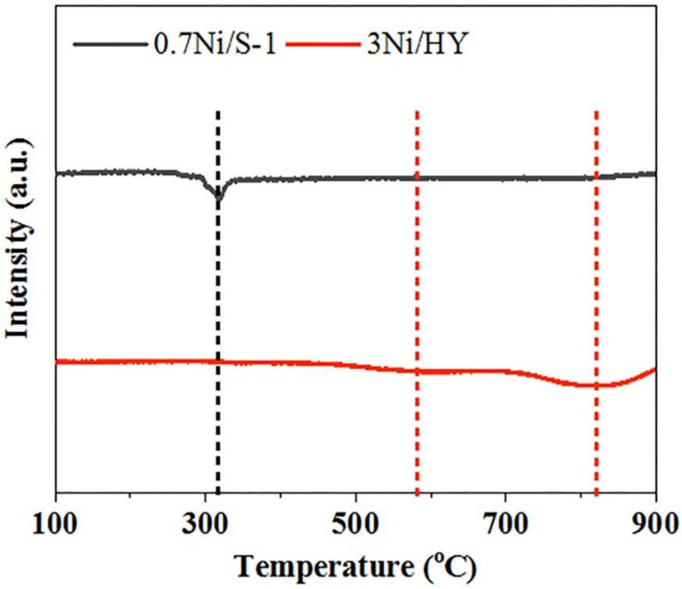

Supplementary Figure. 10 H_2-TPSR results of 3Ni/HY and 0.7Ni/S-1. Reaction condition: 200 mg catalysts, 10% H_2 with He as balance gas.

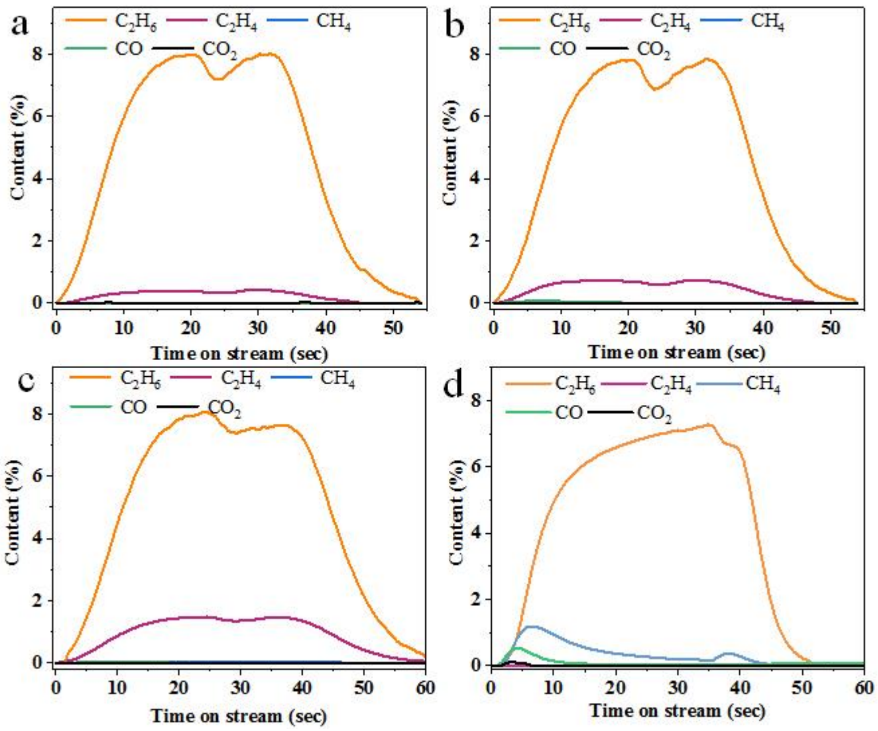

Supplementary Figure 11. Reaction kinetic profile of **a** 1Ni/HY, **b** 2Ni/HY, **c** 3Ni/HY, **d** 0.7Ni/S-1, reaction condition: 600 ºC, 10% C_2H_6 with 30 sec reduction, GHSV = 5100 h^{-1} (15000 h^{-1} for 0.7Ni/S-1).

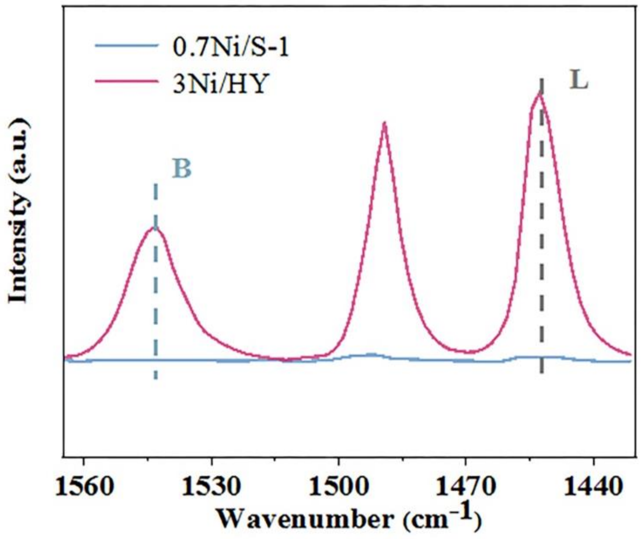

Supplementary Figure. 12 Pyridine adsorption FT-IR spectra of 0.7Ni/S-1 and 3Ni/HY. (B and L in the diagram stand for Brønsted and Lewis acid sites, respectively.)

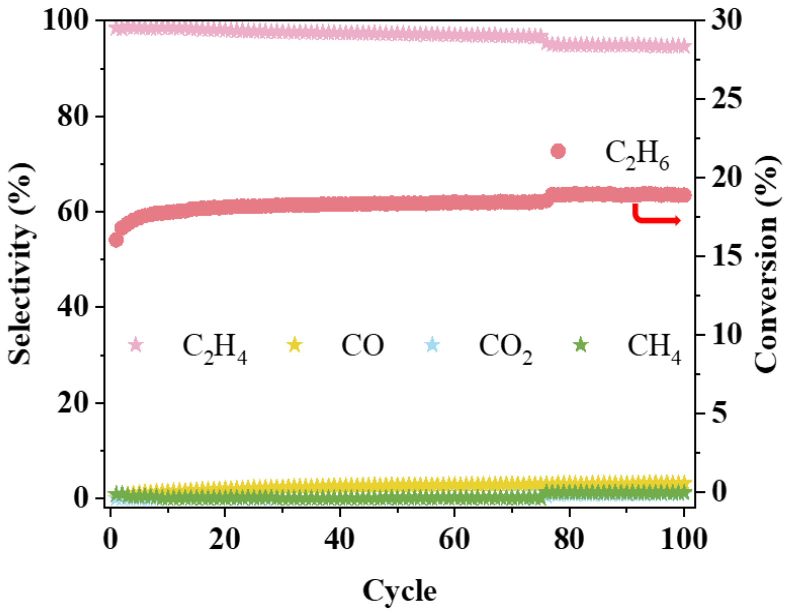

Supplementary Figure. 13 Long-term stability test over the 3Ni/HY during the 108 redox cycles.

The stability test was carried out over 3Ni/HY for 108 cycles at 600 °C. The product component of outlet streams is measured by an online Fourier transform infrared (FTIR) analyzer (Bruker, MATRIX-MG(HR)-01), equipped with a liquid N_2-cooled mercury-cadmium-telluride detector. Prior to each measurement, 25 ml gas cell was heated to 30 °C and the background scans (30 times) was settled. Each measurement data derived from the average of 3 scans of the band 700-4000 cm^{-1}, lasting 0.7 s at a resolution of 1 cm^{-1}. The collected spectra were analyzed for C_2H_6, C_2H_4, CH_4, CO, and CO_2, using software provided (Bruker, MG01). Each species was quantified by adapting their characteristic absorption band to exclude interfering peaks from other species. The ethane conversion (Con) and ethene selectivity (Sel) based on all products (include coking formation and gas phase product calculated by integrating corresponding IR signals) were defined as

$$\text{Con (\%)} = \frac{\int_0^{t_{red}}[y_{C_2H_4}+\frac{1}{2}(y_{CH_4}+y_{CO}+y_{CO_2})]\text{dt} + \frac{1}{2}\int_0^{t_{ox}}(y_{CO,\text{ox}}+y_{CO_2,ox})dt}{\int_0^{t_{red}}[y_{C_2H_4}+y_{C_2H_6}+\frac{1}{2}(y_{CH_4}+y_{CO}+y_{CO_2})]\text{dt} + \frac{1}{2}\int_0^{t_{ox}}(y_{CO,ox}+y_{CO_2,ox})dt} \times 100 \quad (2)$$

$$\text{Sel (\%)} = \frac{\int_0^{t_{red}} y_{C_2H_4}\text{dt}}{\int_0^{t_{red}}[y_{C_2H_4}+y_{C_2H_6}+\frac{1}{2}(y_{CH_4}+y_{CO}+y_{CO_2})]\text{dt}+\frac{1}{2}\int_0^{t_{ox}}(y_{CO,ox}+y_{CO_2,ox})dt} \times 100 \quad (3)$$

Where y_{C2H4}, y_{C2H6}, y_{CH4}, y_{CO2}, and y_{CO} are the volume fraction of C_2H_4, C_2H_6, CH_4, CO_2, and CO in the effluent gas in the reduction step, respectively. In addition, the $y_{CO,ox}$ and $y_{CO2,ox}$ are the volume fraction of CO and CO_2 in the effluent gas in the re-oxidation step, t_{red} and t_{ox} for integration are the time span of the reduction and oxidation step in documented data, respectively (the range of this time is not necessarily equal to feeding time because of the response of the analyzer and dead volume).

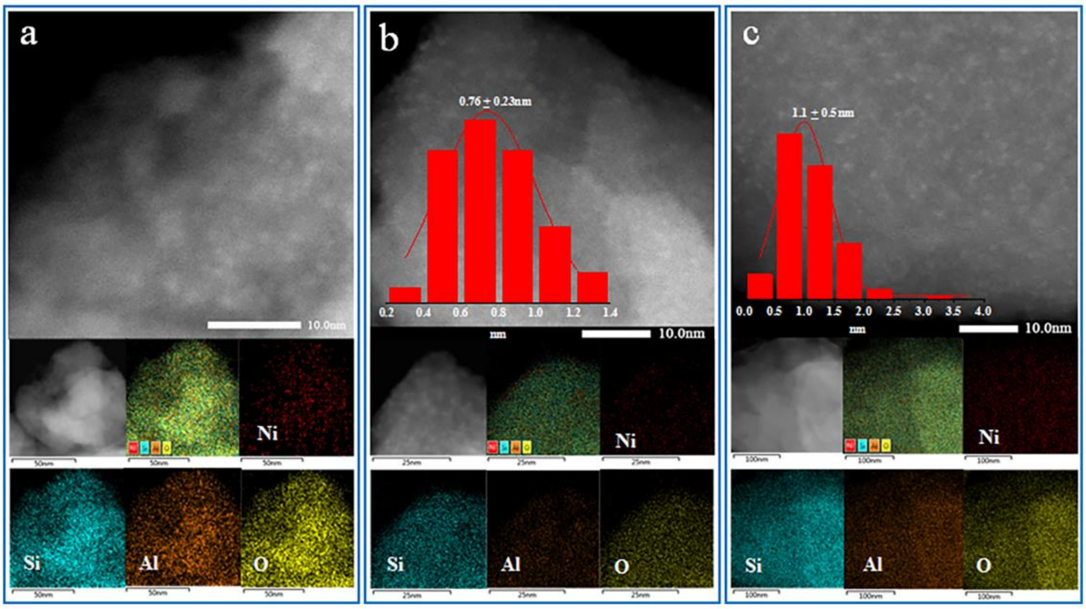

Supplementary Figure. 14 a-c HAADF-STEM images and EDS mappings of the reduced xNi/HY (x=1, 2, and 3), respectively.

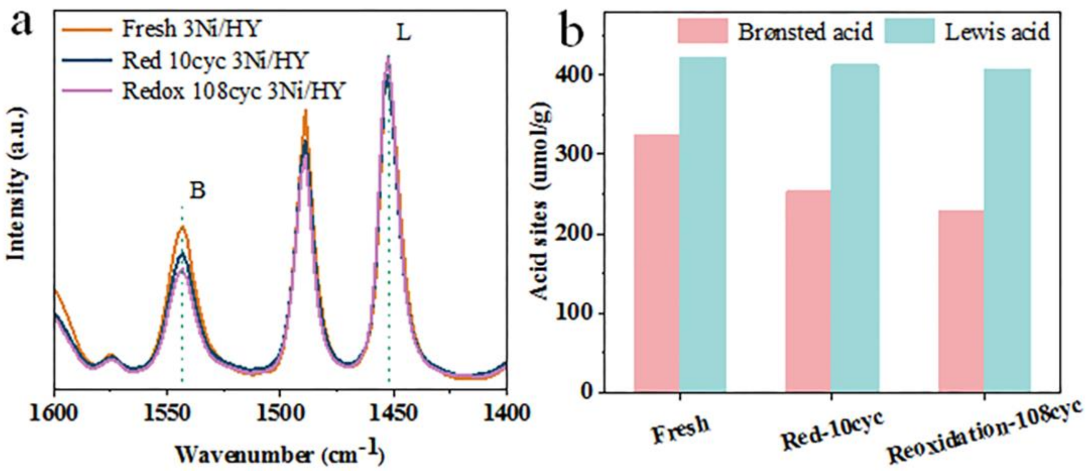

Supplementary Figure. 15 a Pyridine adsorption FT-IR spectra of fresh 3Ni/HY, and after 10th reduction and 108 cycles. **b** Corresponding quantification of Lewis acid sites from FT-IR spectra in **a**. (B and L in the diagram stand for Brønsted and Lewis acid sites, respectively.)

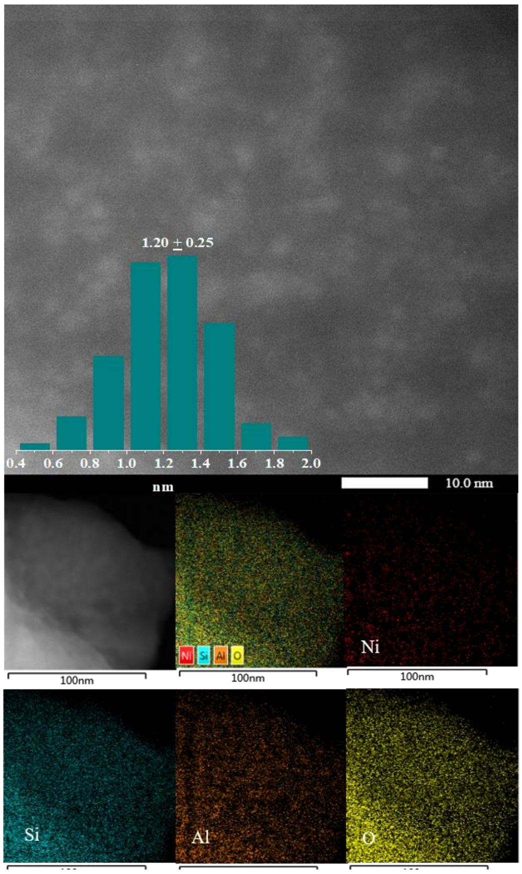

Supplementary Figure. 16 HAADF-STEM image and EDS mapping of the 3Ni/HY after 108 redox cycles.

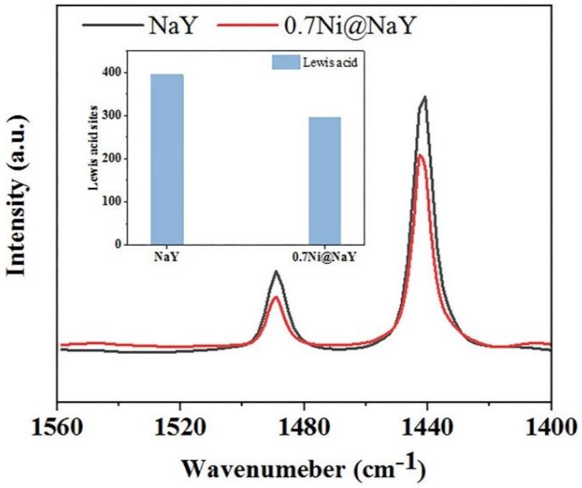

Supplementary Figure. 17 Pyridine adsorbed FT-IR spectra of NaY and 0.7Ni@NaY. Inset: corresponding amount of acid sites of those.

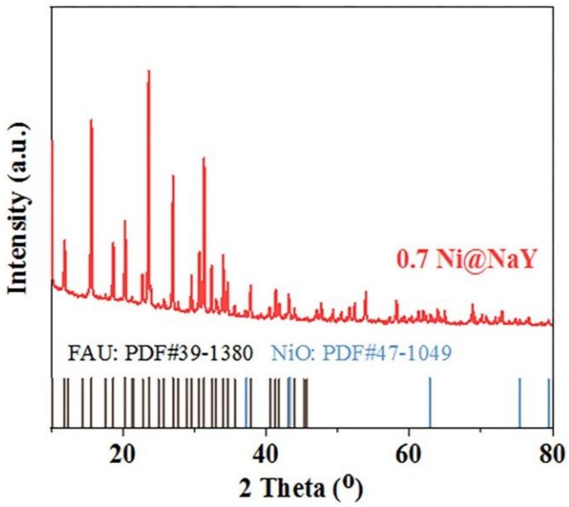

Supplementary Figure. 18 XRD patterns of as-synthesized 0.7Ni@NaY.

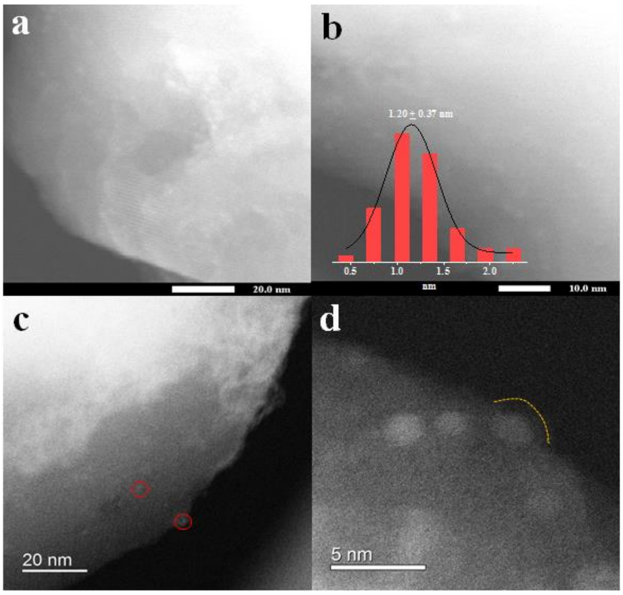

Supplementary Figure. 19 a-b HAADF-STEM images and **c-d** AC-TEM images of the 0.7Ni@NaY.

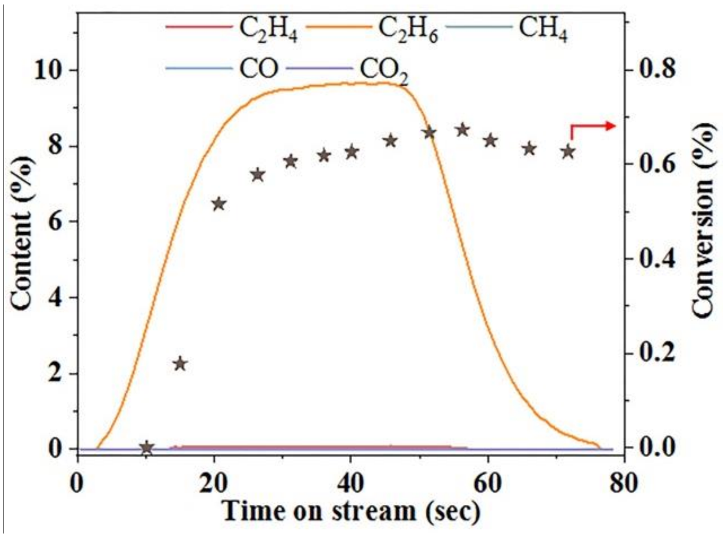

Supplementary Figure. 20 Redox test result of 0.7Ni@NaY, reaction condition: 600 °C, 10% C_2H_6 with 30 sec reduction, GHSV = 5100 h^{-1}.

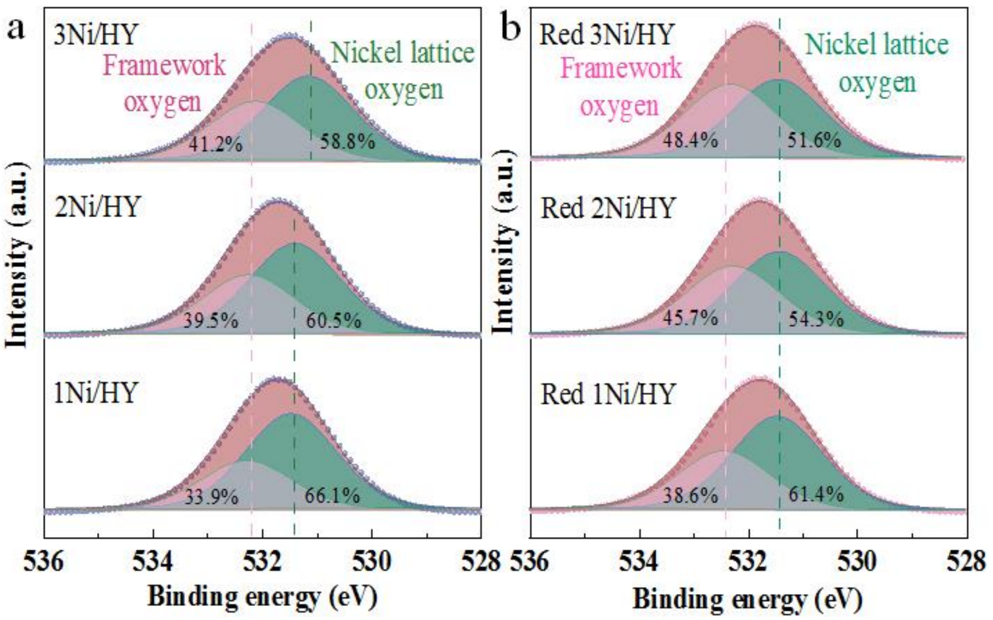

Supplementary Figure. 21 O *1s* XPS spectra of the fresh and reduced xNi/HY samples.

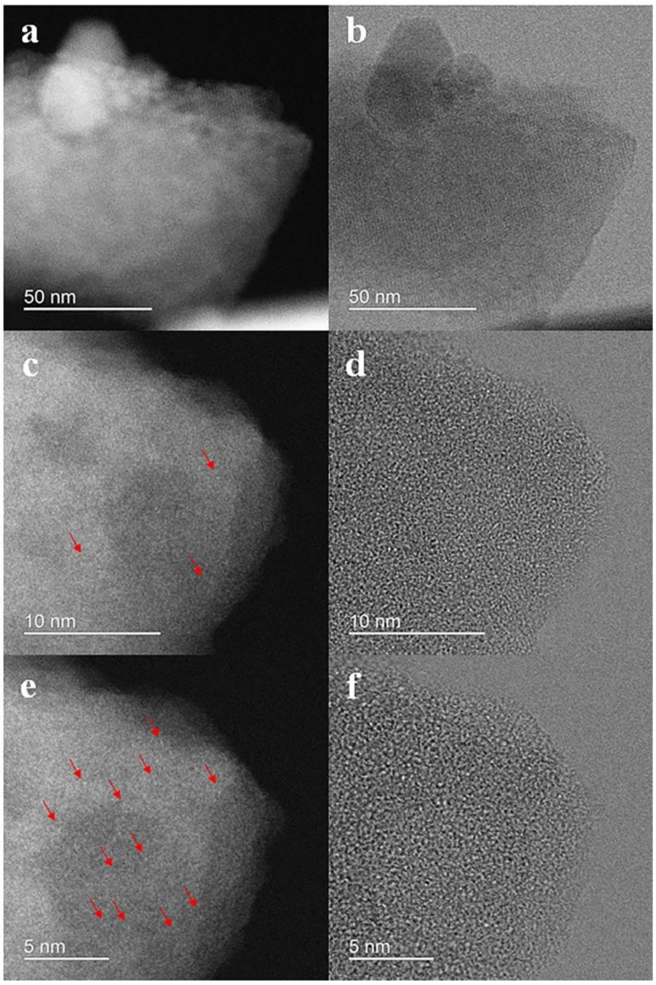

Supplementary Figure. 22 a-f AC-TEM images of the 3Ni/HY-IE.

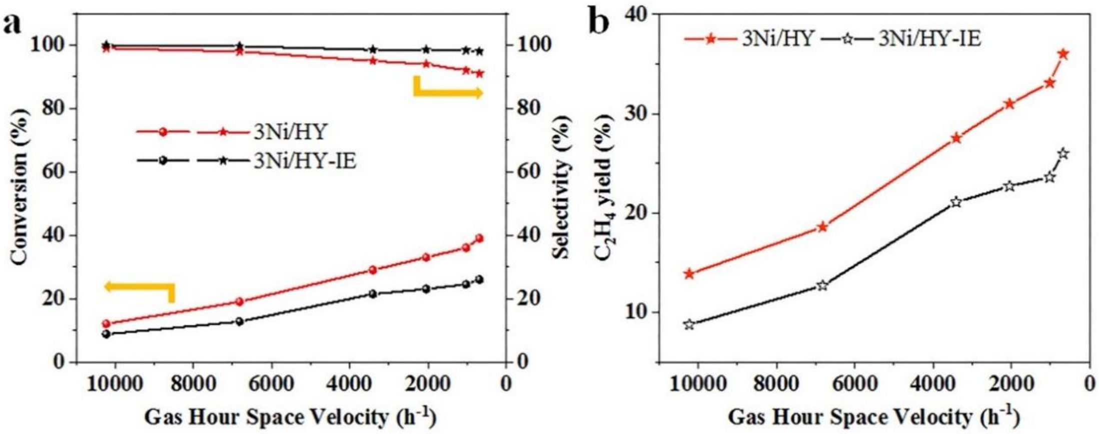

Supplementary Figure. 23 Comparison of **a** C_2H_6 conversion and C_2H_4 selectivity and **b** C_2H_4 yield over 3Ni/HY and 3Ni/HY-IE under different space velocity (600 °C, 5% C_2H_6/He with 30 sec reduction, 500 mg catalysts).

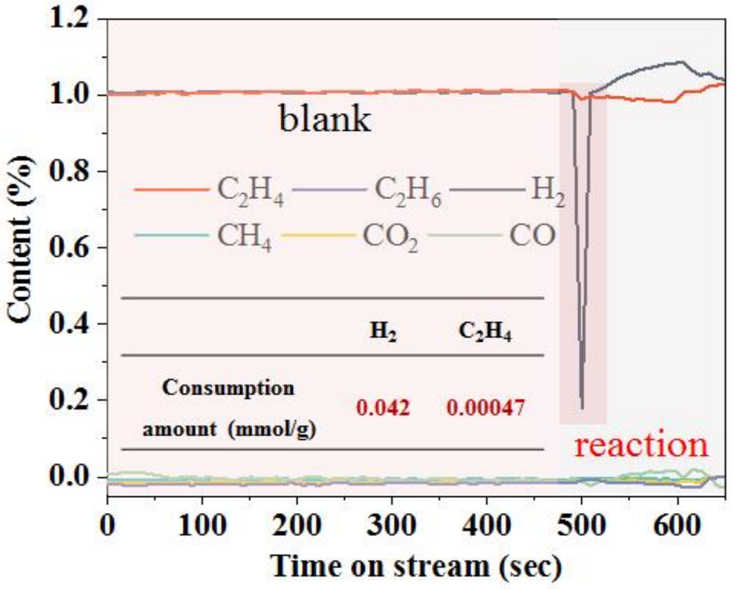

Supplementary Figure. 24 The reaction profile on 3Ni/HY under the co-feeding of C_2H_4 and H_2, reaction condition: 600 ºC, 1% C_2H_4-1% H_2/He at a constant 18000 h^{-1} GHSV. Inset: the consumption amount of H_2 and C_2H_4.

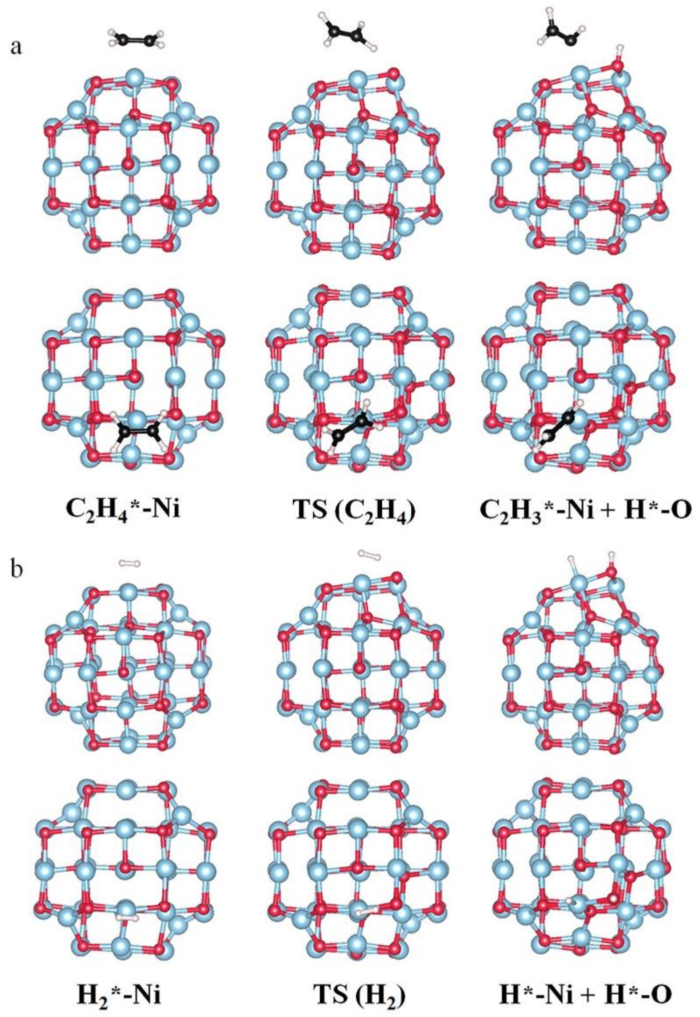

Supplementary Figure. 25 The side and top views for the corresponding geometric structure of reaction intermediate of a) C_2H_4 and b) H_2 dissociation on 1nm NiO nanocluster. C_2H_4*-Ni, TS (C_2H_4) and C_2H_3*-Ni + H*-O stand for C_2H_4 adsorption, TS (transition state) of the C_2H_4 dissociation and dissociation products of C_2H_4, respectively; H_2*-Ni, TS (H_2) and H*-Ni + H*-O stand for H_2 adsorption, TS (transition state) of the H_2 dissociation and dissociation products of H_2, respectively. Color index: Ni, blue; O, red; C, black; H, white.

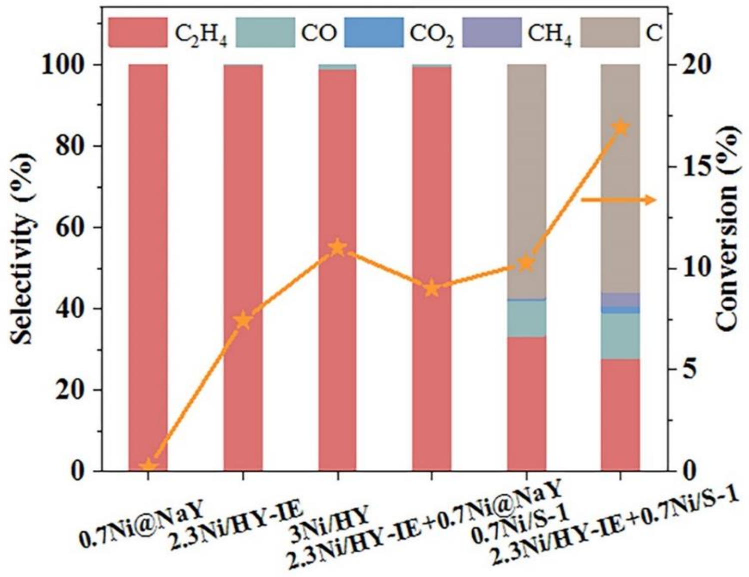

Supplementary Figure. 26 Comparison of redox test results for different Ni species, reaction condition: 600 °C, 5% C_2H_6 with 30 sec reduction, 500 mg catalysts, GHSV = 10200 h^{-1}. Each bed contained 500 mg of catalysts for two double bed tests with the space velocity of 5100 h^{-1}. Specially, 0.7Ni/S-1 and 2.3Ni/HY-IE+0.7Ni/S-1 were operated at higher space velocity with 37500 and 18700 h^{-1}, respectively (C in the legend is corresponding to carbon deposition).

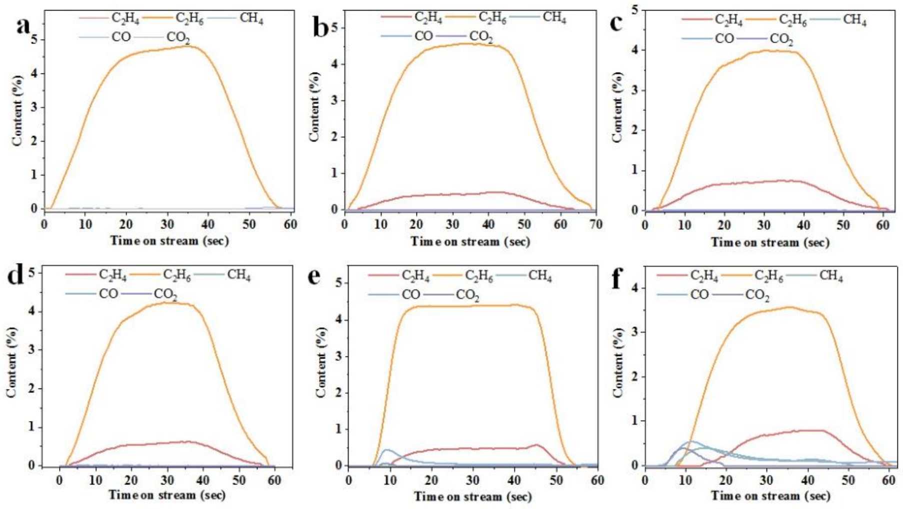

Supplementary Figure. 27 Reaction kinetic profile of **a** 500 mg 0.7Ni@NaY, **b** 500 mg 2.3Ni/HY-IE, **c** 500 mg 3Ni/HY, **d** 500 mg 2.3Ni/HY-IE+500 mg 0.7Ni@NaY for two double bed test **e** 500 mg 0.7Ni/S-1 **f** 500 mg 2.3Ni/HY-IE+500 mg 0.7Ni/S-1 for two double bed test, reaction condition: 600 ºC, 5% C_2H_6 with 30 sec reduction.

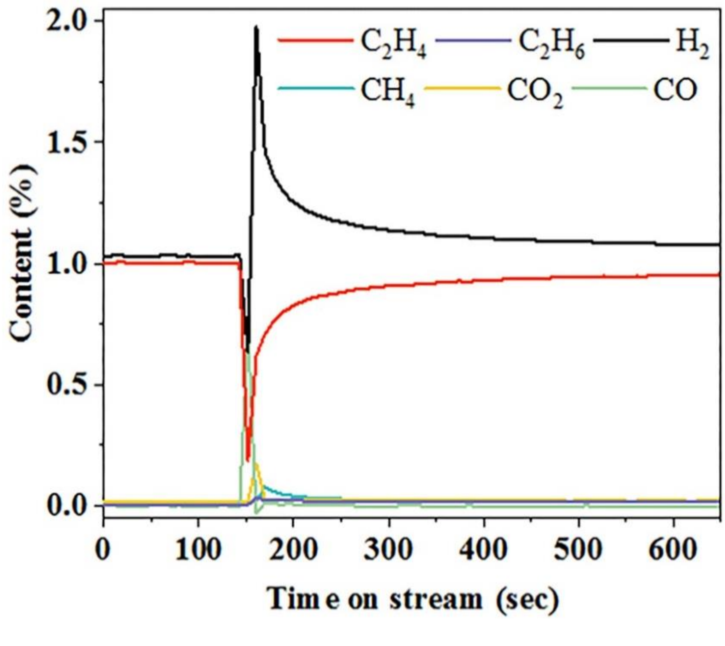

Supplementary Figure. 28 The reaction profile on 0.7Ni/S-1 under the co-feeding of C_2H_4 and H_2, reaction condition: 600 ºC, 1% C_2H_4-1% H_2/He at a constant 18000 h^{-1} GHSV.

Supplementary Table 1. Nominal and actual Ni loadings, and the amount of different Ni species in xNi/HY.

Samples	Nominal Ni loading (wt%)	Actual Ni loading (wt%)[a]	Ni^{2+} LAS (mmol/g)[b]	NiO nanoclusters (mmol/g)[c]
1Ni/HY	0.8	0.8809	0.12	0.029
2Ni/HY	1.6	1.6760	0.19	0.095
3Ni/HY	2.3	2.4670	0.32	0.100
2.3Ni/HY-IE	1.7	1.8100	0.32	/
3Ni/HY-IE	2.3	2.4950	0.43	/
0.7Ni/S-1	0.56	0.5786	/	0.099
0.7Ni@NaY	0.56	0.5810	/	0.100

[a]Determined by ICP-OES analysis.

[b]Determined by pyridine adsorbed IR

[c]Determined by subtracting Ni^{2+} LAS content from the actual Ni loadings

ICP results indicated that actual Ni amount was similar to theoretical one

Supplementary Table 2. Oxygen consumption of xNi/HY during reduction stage.

Samples	1Ni/HY	2Ni/HY	3Ni/HY	0.7Ni/S-1
Oxygen consumption (%)[a]	51.8	31.7	39.2	94.7

[a]Determined by actual oxygen consumption amount in the reduction stage divided by the oxygen capacity of NiO nanoclusters which was obtained by subtracting Ni^{2+} LAS content from the actual Ni loadings

Supplementary Table 2 showed the oxygen consumption (O_C) of xNi/HY and 0.7Ni/S-1 under reaction conditions. O_C is the ratio of oxygen consumption to total theoretical reducible lattice oxygen, which was calculated as O_C (%) =

$$\frac{F_{red}\times\int_0^{t_{red}}[y_{C_2H_4}+y_{CO}+2y_{CO_2}+\frac{1}{2}(3y_{CO}+3y_{CO_2}-y_{CH_4})]\mathrm{dt}}{x\times M/74.69\times 22.4\times 1000}\times 100 \tag{4}$$

Supplementary Table 3. The amount of lattice oxygen associated with Ni species for fresh and reduced xNi/HY samples obtained in O *1s* XPS

Samples	Fresh	Reduced
1Ni/HY	66.1%	61.4%
2Ni/HY	60.5%	54.3%
3Ni/HY	58.8%	51.6%
